# Microbiome and intestinal ischemia/reperfusion injury

**DOI:** 10.3164/jcbn.17-137

**Published:** 2018-05-25

**Authors:** Yuji Nadatani, Toshio Watanabe, Sunao Shimada, Koji Otani, Tetsuya Tanigawa, Yasuhiro Fujiwara

**Affiliations:** 1 Department of Gastroenterology, Osaka City University Graduate School of Medicine, 1-4-3 Asahimachi, Abeno-ku, Osaka City, Osaka 545-8585, Japan

**Keywords:** intestinal ischemia reperfusion injury, microbiome, Toll-like receptors, oxidative stress

## Abstract

Intestinal ischemia/reperfusion injury is a severe disease associated with a high mortality. The mechanisms that cause ischemia/reperfusion injury are complex and many factors are involved in the injury formation process; however, the only available treatment is surgical intervention. Recent studies demonstrated that the intestinal microbiome plays a key role in intestinal ischemia/reperfusion injury and there are many factors associated with intestinal bacteria during the formation of the intestinal ischemia/reperfusion injury. Among the Toll-like receptors (TLR), TLR2, TLR4, and their adaptor protein, myeloid differentiation primary-response 88 (MyD88), have been reported to be involved in intestinal ischemia/reperfusion injury. Oxidative stress and nitric oxide are also associated with intestinal bacteria during the formation of the intestinal ischemia/reperfusion injury. This review focuses on our current understanding of the impact of the microbiome, including the roles of the TLRs, oxidative stress, and nitric oxide, on intestinal ischemia/reperfusion injury.

## Introduction

Many studies have investigated ischemia/reperfusion (I/R) injury in cardiac,^(1)^ cerebral,^(2)^ and hepatic diseases,^(3)^ as well as intestinal injury and transplantation.^(4)^ Among these organs, the intestine is the most sensitive to I/R injury.^(5)^ Acute mesenteric ischemia (AMI) is a typical intestinal I/R injury-related disease that is caused by rapid interruption of blood flow in the mesenteric vessel. AMI occurs in a variety of clinical conditions, including small bowel occlusion and thrombosis of the mesenteric artery, vascular surgery, shock, small bowel transplantation, and trauma.^(6)^ AMI is divided into two types: nonocclusive mesenteric ischemia (NOMI) and occlusive mesenteric arterial ischemia.^(7–9)^

NOMI is an acute mesenteric circulatory disorder that is not caused by organic occlusion of blood vessels. Most cases involve spasm of the superior mesenteric artery (SMA) and the disease easily advances to an irreversible intestinal necrosis because of the difficulty in making a definitive diagnosis.^(10)^ Usually, patients with NOMI are critically ill with severe heart failure, hemodialysis, aortic insufficiency, septic shock, or myocardial infarction and the mortality rate exceeds 50%.^(10–15)^ In the primary stage of NOMI, the intestine is damaged by the interruption of blood flow. Although the primary injury could be repaired by re-establishing the normal mesenteric blood supply, reperfusion could further worsen the initial intestinal damage. This is known as intestinal I/R injury. Mortality rates are especially high when the intestinal I/R injury progresses to shock, multiple organ failure, and sepsis.^(8,16)^

Guidelines of the European journal of trauma and emergency surgery recommend that broad spectrum antibiotics should be administered because bacterial translocation is an early event in the progress of AMI.^(17)^ However, no clinical studies have shown the efficacy of prophylactic antibiotics in patients with acute intestinal I/R, and the bacteria responsible for the disease severity and prognosis have not yet been identified.

Occlusive mesenteric arterial ischemia is subdivided into acute mesenteric arterial embolism and acute mesenteric arterial thrombosis. These diseases are also severe conditions. However, these disease states are primarily ischemic injuries rather than I/R injuries, because embolisms and thrombosis rarely occur naturally.

Because of the disease severity and its prevalence, there are many models and studies targeting intestinal I/R injury. However, the disease mechanisms are not completely understood and there is little useful treatment other than surgical intervention and supportive care. A better understanding of the pathogenesis of intestinal I/R injury is needed to develop new treatments and improve the prognosis. This review focuses on the recent advances in the mechanisms of intestinal I/R and describes the role of the intestinal microbiome during intestinal I/R injury.

## Mechanisms of Ischemia/Reperfusion Injury

The mechanisms of the I/R injury are complex and many factors are involved in the injury formation process. Therefore, many experimental animal models have been established to evaluate the pathogenesis and mechanisms of intestinal I/R injury. Complete ischemia by temporary vascular occlusion with vascular clips or permanent vascular occlusion by ligation of the SMA in rodents are the most commonly used methods used for studying intestinal I/R injury. In most studies, the small intestine was examined after 60 min of ischemia with a reperfusion time of 90–120 min.^(18–20)^

Animal studies indicated that intestinal I/R injuries occur after two events: ischemic injury and reperfusion injury. Ischemic injury is caused by microcirculatory flow disorders. In this stage, the tissue of the small intestine is injured mainly due to hypoxemia. Resumption of blood flow after the original ischemic event further exacerbates the intestinal injury; however, it is necessary for intestinal epithelial cell survival.^(21,22)^ This injury is known as the reperfusion injury.

Mucosal barrier function is destroyed and vascular permeability is increased during the formation of the I/R injury.^(23)^ The increased vascular permeability allows the activation and adhesion of inflammatory cells. These inflammatory cells release reactive oxygen species (ROS), proinflammatory chemokines, and protein kinases.^(5)^ Representative induced molecules include inducible nitric oxide synthase (iNOS), cyclooxygenase-2 (COX-2), tumor necrosis factor α (TNF-α), interleukin (IL)-1, IL-6, prostaglandin, intracellular adhesion molecule, and E-selectin.^(24–28)^

Additionally, the intestinal barrier dysfunction causes bacteria to infiltrate the intestinal mucosa and submucosa after I/R injury.^(29)^ This bacterial translocation stimulates inflammatory cells and induces the inflammatory response while releasing proinflammatory chemokines and protein kinases.^(23)^ The bacteria that penetrate the mucosa reach other organs through the blood circulation. João *et al.*^(29)^ demonstrated that 99mTc-labeled bacteria that was gavaged before I/R treatment were detected in the serum, lung, liver and mesenteric lymph nodes after mesenteric I/R injury in a time dependent manner. Recent studies indicated that the bacteria played a key direct or indirect role in the intestinal I/R injury.

## Intestinal Microbiome

A large number of bacteria exist in the human gastrointestinal tract, especially in the colon. Recent studies revealed that these intestinal bacteria, collectively called the microbiome, play important roles in maintaining the gut mucosal barrier and intestinal immune system.^(30)^ The microbiome interacts with human intestinal environments and modulates host physiological functions such as food digestion and metabolism of nutrition,^(31,32)^ immunomodulation,^(33)^ resistance to pathogens,^(34)^ and so on. Therefore, the gut microbiome attracted attention, because studies have reported that many diseases, including obesity,^(31,32)^ diabetes,^(35)^ liver cancer,^(36)^ IBS,^(37)^ IBD,^(38)^ and non-steroidal anti-inflammatory drug (NSAID)-induced small intestinal damage,^(39)^ were associated with abnormalities of the gut microbiome, called dysbiosis.

It is also thought that dysbiosis is involved in the pathological condition in intestinal I/R injury. Souza *et al.*^(40)^ reported that intestinal I/R injury was inhibited in germ-free mice. Yoshiya *et al.*^(41)^ demonstrated that depletion of gut commensal bacteria by oral administration of a broad-spectrum antibiotic cocktail for four weeks attenuated intestinal I/R injury. Watanabe *et al.*^(19)^ demonstrated that oral administration of ampicillin significantly reduced intestinal I/R injury in mice. In contrast, Chen *et al.*^(42)^ demonstrated that intestinal microbiome depletion by oral administration of broad-spectrum antibiotics exacerbated intestinal I/R injury in mice.

Collectively, these results strongly suggest that intestinal bacteria may mediate I/R injury, although some conflicting results have been reported. However, our knowledge about bacterial involvement in the damage is very limited. No study has discussed in detail the small intestinal microbiome change during I/R injury, although one report demonstrated that the colon microbiome changed before and after intestinal I/R injury.^(43)^ Intensive studies using next generation sequencing could lead to the identification of bacteria responsible for the induction of the I/R damage, and development of useful agents against this damage.

Some reports suggested that probiotics, which regulate the intestinal microbiome, may be candidates for use as therapeutic agents against intestinal I/R injury. Several studies demonstrated that pretreatment with *Lactobacillus plantarum* ameliorated rodent small intestinal I/R injury by decreasing inflammatory cytokines and preventing intestinal barrier dysfunction.^(23,44)^ Salim *et al.*^(45)^ demonstrated that VSL#3, which is a probiotic preparation of eight live freeze-dried bacterial species including *Lactobacillus plantarum*, reduced intestinal I/R injury and inflammation. Thus, some probiotic such as *Lactobacillus plantarum* suppress intestinal inflammation and play protective roles against intestinal I/R injury. In other intestinal injury models, viable lactobacillus strains have been also reported to possess antimicrobial activity.^(46,47)^ Additionally, Ménard *et al.*^(46)^ demonstrated that lactic acid bacterial strains and their metabolites inhibited the binding of lipopolysaccharide (LPS) to THP-1 cells, and decreased NF-κB nuclear translocation. Although the mechanisms by which these probiotic bacteria ameliorate intestinal I/R injury is still unclear, bacteriostatic and immunosuppressive actions may be involved in the prevention of the damage by the bacteria. Even though probiotics are effective against I/R injury in animal models, it is not realistic to use them as therapeutic agents for treating intestinal I/R injury in humans, because probiotics are usually prescribed orally. Therefore, administering probiotics alone or in combination with antibiotics could be candidate agents for the prophylaxis of intestinal I/R injury in high-risk patients, such as those who will undergo small bowel surgery. Clinical trials to evaluate the prophylactic efficacy of probiotics should be conducted to prove their usefulness.

## Toll-like Receptors

A representative receptor that recognizes the constituent molecules of bacteria and contributes to the homeostasis of the intestinal environment is the Toll-like receptor (TLR). TLRs play important roles in the innate immune system. Researchers have identified 10 TLRs in humans and 12 in mice. Although specific ligands are recognized by each TLR,^(48)^ they generally recognize pathogen-associated molecular patterns and danger-associated molecular patterns.^(49)^ The TLR signaling pathway activates inflammatory responses, such as the secretion of NF-κB and TNF-α. Among these TLRs, previous reports suggested that TLR2 and TLR4 play important roles in intestinal I/R injury. The reported role of TLR2 and TLR4 in intestinal I/R injury is controversial; however, majority of the studies conducted in pathological models other than those of intestinal inflammation have reported that the TLR2 and TLR4 signaling pathways induce inflammation.

TLR2 is a membrane surface receptor that is activated by bacterial peptidoglycans, which are highly expressed on the outer membranes of gram-positive bacteria, and in fungal substances. TLR2 is expressed on microglia and inflammatory cells, such as monocyte/macrophage, dendric cells, B lymphoceles, and T lymphoceles. In general, the TLR2 signaling pathway leads to production of TNF-α and NF-κB.^(48)^

Several studies in a TLR2 knockout mouse model indicated that the role of TLR2 in intestinal I/R injury is controversial. Several studies reported that small intestinal injury after I/R treatment in TLR2 knockout mice was increased compare to that in wild-type mice.^(18,50)^ In this study, secretory immunoglobulin A, which is a protective factor in intestinal I/R injury, was dependent on TLR2 activity and TLR2 knockout mice had more inflammatory cytokines in the small intestine. In contrast, in our study, TLR2 knockout mice exhibited less severe I/R injury than wild-type mice with decreasing TNF-α mRNA expression.^(20)^ This study suggested that TLR2 mediated intestinal I/R injury via induction of inflammatory mediators. There is a possibility that the balance between the TLR2-related offensive factor and defensive factor may have contributed to the intestinal I/R injury. In addition, the results may be different if the study conditions change (Table 1).

TLR4 is also a membrane surface receptor that is activated by the LPS of gram negative bacteria. Similar to that by the TLR2 signaling pathway, TLR4 activation induced production of TNF-α and NF-κB.^(48,51)^ Some reports using TLR4 knockout mice suggested that TLR4 was protective against intestinal I/R injury.^(18)^ Chen *et al.*^(42)^ also suggested that administration of LPS, a representative TLR4 ligand,^(52)^ decreased intestinal I/R injury by preventing an increase in intestinal permeability thorough TNF-α signaling. Contrary to these studies, some reports suggested that the TLR4 signaling pathway is the aggravating factor against intestinal I/R injury.^(53–55)^ Pope *et al.*^(54)^ demonstrated that TLR4 deficiency attenuated intestinal I/R injury via reducing complement activation and Moses *et al.*^(55)^ suggested that TLR4-mediated COX-2 expression increased intestinal I/R injury. Additionally, some reports suggested that high mobility group box 1, a TLR4 ligand,^(56,57)^ was an aggravating factor for intestinal I/R injury.^(58,59)^ Because there are many types of TLR4 ligands, the effect of the TLR4 signaling pathway may be different depending on the activating ligand. However, there is no doubt that the TLR4 signaling pathway plays an important role in intestinal I/R injury (Table 1).

Slone *et al.*^(60)^ reported that the TLR9 pathway was not related to intestinal I/R injury and there have been few reports regarding other TLRs. It is likely that other TLRs play only minor roles in intestinal I/R injury.

Myeloid differentiation primary response 88 (MyD88) is an adapter protein used by all TLRs, except TLR3. During activation of the TLR2 or TLR4 signaling pathway, MyD88 combines an N-terminal death domain with a C-terminal Toll/IL-1 receptor domain that serves to anchor the molecule to the corresponding domain of TLR2 or TLR4. This signaling pathway activates NF-κB.^(48,61)^ We previously reported that MyD88 plays a protective role in intestinal I/R injury via induction of COX-2.^(19)^ In contrast, Wang *et al.*^(58)^ reported blocking MyD88 using anti-MyD88 antibody to ameliorate the mice intestinal I/R injury with reduced NF-κB protein expression. The role of MyD88 reported in these studies was contradictory, similar to the studies regarding the role of TLR2 and TLR4, which are upstream of MyD88, in intestinal I/R injury (Table 1).

The role of TLRs in I/R injury has also been investigated in other organs, such as the heart, brain, kidney, and liver.^(62)^ In cardiac, cerebral, renal, and liver I/R injury, it has been reported that among the TLRs, TLR2, TLR4, and MyD88 are receptors that are mainly involved in pathological conditions. Interestingly, most studies have indicated that TLR2, TLR4, and MyD88 are exacerbating factors for I/R injury in other organs, unlike the studies conducted in the intestine.

In studies regarding the small intestine in other pathological rodent models, TLR2, TLR4, and MyD88 are often described as factors that exacerbate inflammation. A typical disease involving intestinal bacteria in the disease state is NSAID-induced small intestinal injury. Most studies, including our study, have reported that the TLR4 and MyD88 signaling pathways are aggravating factors for NSAID-induced small intestinal injury.^(56,63,64)^

Contrary to studies conducted in other organs, the role of TLRs in intestinal I/R injury is controversial. One of the reasons for this is that bacteria constantly exist in the gastrointestinal tract, unlike other organs. Some papers suggested that the TLR signaling pathway induced by commensal bacteria has a beneficial role. Rakoff-Nahoum *et al.*^(65)^ demonstrated that the long-term depletion of commensal bacteria, as well as genetic deletion of MyD88, modulates the expression of several molecules such as TLRs and heat-shock proteins, which play important roles in maintaining intestinal homeostasis. They suggested that the recognition of commensal bacteria by TLRs plays a beneficial role in the control of intestinal epithelial homeostasis and protection from direct injury.

Thus, the balance between the beneficial and detrimental effects of the TLR pathway may be disturbed in the intestines of TLR and MyD88 knock out mice, in whom the TLR signaling was absent. This imbalance may lead to a high susceptibility to intestinal I/R injury in such mice, resulting in controversial results among experimental studies using TLR knockout mice and those using the antagonists or agonists of the corresponding receptors. Therefore, studies using conditional knockouts of TLRs and MyD88, or the agonists and antagonists against TLRs and MyD88 are necessary to clarify the precise role of the TLR and MyD88 pathways in the development and progression of intestinal I/R.

## Oxidative Stress

Many studies have demonstrated that oxidative stress caused by ROS and free radicals, including superoxide anion radicals (O_2_^•−^), hydrogen peroxide (H_2_O_2_), singlet oxygen (^1^O_2_), and hydroxyl radical (^•^OH), affects the intestine during reperfusion of the ischemic small intestine.^(66–69)^ An important source of these ROS and free radicals are neutrophils and macrophages. These inflammatory cells release ROS and free radicals during respiratory bursts against invading bacteria. Although this production of ROS and free radicals is designed to sterilize invading bacteria, excessive production leads to local tissue injuries because of the excessive inflammatory reaction.

Xanthine oxide (XO) metabolism is another important source of free radicals during I/R injury. In ischemic situations, XO is generated from xanthine dehydrogenase by Ca^2+^-dependent proteases and generates singlet oxygen and hydrogen peroxide by catalyzing the oxidation of xanthine and hypoxanthine into uric acid.^(70)^ This XO system can produce large amounts of ROS during the formation of the I/R injury. Inhibition of XO by the purine analog, allopurinol, protects the intestine from I/R injury.^(71–73)^

Prevention of oxidative stress can be useful for the treatment of intestinal I/R injury. Many studies demonstrated that heme oxygenase (HO)-1 plays a protective role, especially in I/R injury, by preventing oxidation.^(74–76)^ HO-1 is an enzyme that catalyzes the degradation of heme to carbon monoxide, biliverdin, and free iron. Carbon monoxide is not an antioxidant,^(77)^ but it plays a protective role in intestinal I/R injury via anti-inflammatory and anti-apoptotic properties.^(24,26)^ Biliverdin and its metabolite bilirubin are also antioxidants. Some reports demonstrated that the administration of bilirubin ameliorated the intestinal I/R injury by reducing inflammatory cytokines.^(78,79)^

Many other antioxidants have also been reported to have protective effects against intestinal I/R injury (i.e., melatonin, *N*-acetylcysteine, dimethyl sulfoxide, superoxide dismutase, and vitamin C).^(5,72,80–91)^

Therefore, oxidative stress plays important role in intestinal I/R injury. Although ROS and super oxide from inflammatory cells are necessary to maintain intestinal homeostasis and protect against invading bacteria, excessive production of ROS and super oxide cause tissue injury. Antioxidants, which eliminate excess ROS and super oxide, may be candidates for the treatment of intestinal I/R injury.

## Nitric Oxide

Nitric oxide (NO) is an important mediator of physiological and pathological processes. Many studies have demonstrated that NO regulated intestinal blood flow by relaxing the vascular endothelium.^(92,93)^ Increasing the blood flow allows inflammatory cytokines to be carried away and improves local inflammation. However, sometimes, increased blood flow may carry bacteria that passed through intestinal epithelial barrier to another organ and cause remote organ failure.

NO plays another important role related to oxidative stress in intestinal I/R injury. NO is produced from the guanidine group of l-arginine by NO synthase (NOS). Four major isoforms of NOS, including neuronal NOS (nNOS), endothelial NOS (eNOS), iNOS, and mitochondrial NOS (mtNOS), have been identified and all four isoforms are present throughout the gastrointestinal tract.^(94–97)^ iNOS is induced and produces NO after stimulation by proinflammatory cytokines, although nNOS and eNOS are naturally present.^(94,98,99)^ mtNOS is a Ca^2+^-dependent NOS subtype found in the inner membrane of the mitochondria.^(97)^ Although NO produced through constitutive NO synthase (nNOS and eNOS) can be an important protective molecule against intestinal I/R injury, over-production of NO through iNOS may aggravate inflammation.^(96,100–102)^

A more likely explanation for the protective effect of nNOS and the NO produced thereby is that they act as antioxidants during the inflammatory response.^(71)^ Additionally, damage and loss of function of nNOS immunopositive myelin neurons may be fundamental to bowel movement disorders, suggesting that NO produced by nNOS is protective against metabolic injury.^(103–105)^

In contrast, NO is involved in the inflammatory cascade by radical-mediated mechanisms in overproduction situations. NO captures and eliminates other free radicals. However, at the same time, another strong free radical called peroxynitrite (ONNO^−^) is generated.^(106)^ Peroxynitrite can be protonated to the highly cytotoxic peroxynitrous acid (ONOOH) and cause DNA breakage and cell injury.^(107)^

Therefore, regarding intestinal I/R injury, the results of the previous studies regarding the influence of NOS are controversial and depended on which NOS was primarily involved. Additionally, Naito *et al.*^(108)^ reported that NO produced by iNOS exacerbated intestinal I/R injury by lipid peroxidation and administration of the selective iNOS inhibitor ameliorated the I/R injury in rats. In contrast, Margaritis *et al.*^(71)^ reported that oral administration of the nonselective NOS inhibitor, *N*^G^-nitro-l-arginine methyl ester, aggravated rat intestinal I/R injury with increasing neutrophil infiltration.

NO has many other beneficial effects during the formation of intestinal I/R injury. NO can induce apoptosis and necrosis. High levels of NO-induced apoptosis in I/R injury and an inhibitor of iNOS led to a decrease in NO production and subsequent intestinal apoptosis.^(96,109)^ NO also reduced neutrophil infiltration into the gastrointestinal tract in response to acute inflammation and NO inhibition exacerbated leukocyte recruitment.^(96)^ Therefore, there is no doubt that NO and various NOSs play important roles in intestinal I/R injury. However, because NO has both protective and inductive effects in intestinal I/R injury, whether an NO inhibitor or stimulator can be used as a treatment method should be carefully considered.

## Conclusion

**Fig. 1:**
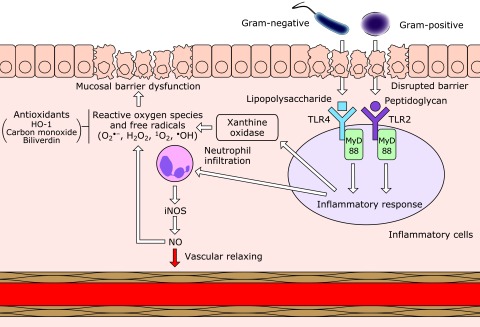
Schematic showing how gut microbiome mediates ischemia/reperfusion injury in the intestine. TLR, Toll-like receptor; MyD88, myeloid differentiation primary-response 88; iNOS, inducible nitric oxide synthase.

Previous findings strongly indicate that the gut microbiome mediates intestinal I/R injury; it is thought to use TLRs to induce the damage (Fig. 1). Animal studies demonstrated that both TLR4, a receptor of LPS of gram-negative bacteria, and TLR2, a receptor of bacterial peptidoglycans, which are highly expressed in gram-positive bacteria, play crucial roles in intestinal I/R injury, suggesting the involvement of multiple bacterial species in the damage. In addition to animal studies to investigate microbial characteristics and those using conditional knockouts of TLRs or specific agonists or antagonists against TLRs, clinical studies evaluating the efficacy of antibiotics should be urgently performed to confirm the involvement of the bacteria in intestinal I/R injury. Metagenomic analyses of human samples could help identify the bacteria responsible for intestinal I/R injury.

## Figures and Tables

**Table 1:** *In vivo* studies on the role of TLRs and MyD88 in I/R injury

Publications	TLRs studied	Beneficial or unbeneficial against intestinal I/R injury	Findings
Tatum, J Pediatr Surg	TLR2 TLR4	beneficial	Neonatal mice deficient in TLR4, either alone or in concert with TLR2, were more susceptible to intestinal mucosal damage.
Watanabe, PLoS One	TLR2	unbeneficial	TLR2^−/−^ mice exhibited less severe mucosal injury and decreased MPO and TNF-α and ICAM-1 mRNA expression.
Aprahamian, Pediatr Crit Care Med	TLR2	beneficial	In TLR2^−/−^ mice, intestinal injury scores increased and the expression of IFN-γ, IL-4, and IL-6 mRNA decreased.
Chen, Shock	TLR4	beneficial	Lipopolysaccharide, a TLR4 ligand, decreased mesenteric I/R injury-induced gut damage through TNF-α signaling.
Zhu, Oncotarget	TLR4	unbeneficial	TLR4 mutation suppressed histological injuries and reduced cytokine expression in the intestine (TNF-α, IL-6, IL-1β, and NF-κB).
Pope, Mol Immunol	TLR4	unbeneficial	TLR4-deficient mice sustained less damage and inflammation after I/R than wild-type mice.
Moses, J Leukoc Biol	TLR4 MyD88	unbeneficial	The absence of TLR4 or MyD88 attenuated local mucosal damage and significantly decreased cytokine and eicosanoid secretion, including PGE_2_ production.
Wang, World J Gastroenterol	TLR4 MyD88	unbeneficial	Blocking HMGB1 and MyD88 reduced the levels of inflammatory cytokines (NF-κB, p65, and TNF-α) in serum.
Kojima, J Surg Res	TLR4	unbeneficial	Anti-HMGB1 antibody treatment significantly reduced the damage and improved the 48-h survival rates.
Slone, Am J Clin Exp Immunol	TLR9	no effect	TLR9 is not required for I/R-induced injury or inflammation of the intestine.
Watanabe, Am J Physiol Gastrointest Liver Physiol	MyD88	beneficial	The MyD88 signaling pathway inhibited I/R injury in the small intestine by inducing COX-2 expression.

TLR, Toll-like receptor; MyD88, myeloid differentiation primary response 88; I/R, ischemia/reperfusion; TNF, tumor necrosis factor; MPO, myeloperoxidase; ICAM, intercellular adhesion molecule; IFN, interferon; IL, interleukin; HMGB, high mobility group box; COX, cyclooxygenase.
